# Full Genome Sequence of a Bovine Enterovirus Isolated in China

**DOI:** 10.1128/genomeA.00620-14

**Published:** 2014-06-26

**Authors:** Xiao-wei Peng, Hao Dong, Qing-min Wu, Yan-li Lu

**Affiliations:** College of Veterinary Medicine, China Agricultural University, Beijing, People’s Republic of China

## Abstract

We report the full genome sequence of an isolate of bovine enterovirus type B from China. The virus (BEV-BJ001) was isolated from Beijing, China, from fecal swabs of cattle suffering from severe diarrhea. This genome sequence will give useful insight for future molecular epidemiological studies in China.

## GENOME ANNOUNCEMENT

Bovine enteroviruses (BEVs) are under the genus *Enterovirus*, family *Picornaviridae*. Bovine enteroviruses are mainly observed in vertebrates’ intestinal tract, where pathogenicity is not obvious, but usually cause mild or asymptomatic infection, and sometimes induce clinical syndromes with significant symptoms (1–3). Bovine enteroviruses have been classified into 2 serotypes, which are currently within the species *Bovine enterovirus* of the genus *Enterovirus* (4). Recently, isolates were re-classified by phylogenetic analysis and 2 BEV clusters were revealed, one of which contains two and the other three geno-/serotypes (5).

In this study, 12 pairs of primers were designed, with several overlapped nucleotides in each fragment. Total RNA was extracted using the RNA-out kit (AndiBio) according to the manufacturer’s protocol. First-strand cDNA was synthesized with the random primer RPAdP and Moloney murine leukemia virus (MMLV) reverse transcriptase (Promega), followed by PCR ampliﬁcation with random primers RPAP and ExTaq DNA polymerase (TaKaRa). The genome terminus sequences were determined by 3′ and 5′ terminus rapid amplification of cDNA ends (RACE) according to the manufacturer’s instructions (TaKaRa Biotech Co., Ltd.). Vector NTI advance 10 was used to assemble the fragments to obtain a complete genome sequence. To check the genotyping of the sample, phylogenetic analysis was performed with MEGA 4 software.

The sequence generated from 12 overlapping fragments of sample BEV-BJ001 covered the entire length of the 7,430 nucleotides (nt), including complete 5′ untranslated region (5′UTR) and 3′UTR with 35-nt poly(A) tails. The single open reading frame (ORF) of BEV-BJ001 is located between nucleotides 824 and 7324, encoding a putative polyprotein of 2,166 amino acids. The genome length of BEV-BJ001 is similar to those (7,426 to 7,450 nt) of other BEV-B strains sequenced before.

Comparison of predicted amino acid sequences representing the poly-protein from several enteroviruses in the NCBI database by using Clustalx 1.83 and rooted by using porcine teschovirus 1 (PTV-1) demonstrated that BEV-BJ001 has 21% to 94% identity with representatives of several enteroviruses, with genotype 2 of the BEV-B cluster having the highest homology (94%). According to phylogenetic analysis, BEV-BJ001 was more closely related to BEV strain wye3A, which has been typed as genotype 2 of BEV-B, than to BEV-A or other enteroviruses. The same results were obtained from comparison of full-length nucleotide sequences.

### Nucleotide sequence accession number.

The complete genome sequence of BEV strain BEV-BJ001 has been deposited in GenBank under the accession no. HQ663846.
